# A rhodium/silicon co-electrocatalyst design concept to surpass platinum hydrogen evolution activity at high overpotentials

**DOI:** 10.1038/ncomms12272

**Published:** 2016-07-22

**Authors:** Lili Zhu, Haiping Lin, Youyong Li, Fan Liao, Yeshayahu Lifshitz, Minqi Sheng, Shuit-Tong Lee, Mingwang Shao

**Affiliations:** 1 Institute of Functional Nano and Soft Materials (FUNSOM), Jiangsu Key Laboratory for Carbon-Based Functional Materials and Devices, Soochow University, Suzhou, Jiangsu, 215123, China; 2 Materials Science and Engineering Department, Technion, Israel Institute of Technology, Haifa 3200003, Israel

## Abstract

Currently, platinum-based electrocatalysts show the best performance for hydrogen evolution. All hydrogen evolution reaction catalysts should however obey Sabatier's principle, that is, the adsorption energy of hydrogen to the catalyst surface should be neither too high nor too low to balance between hydrogen adsorption and desorption. To overcome the limitation of this principle, here we choose a composite (rhodium/silicon nanowire) catalyst, in which hydrogen adsorption occurs on rhodium with a large adsorption energy while hydrogen evolution occurs on silicon with a small adsorption energy. We show that the composite is stable with better hydrogen evolution activity than rhodium nanoparticles and even exceeding those of commercial platinum/carbon at high overpotentials. The results reveal that silicon plays a key role in the electrocatalysis. This work may thus open the door for the design and fabrication of electrocatalysts for high-efficiency electric energy to hydrogen energy conversion.

## Abstract (web summary)

Design of hydrogen evolution catalysts needs to balance the hydrogen adsorption energy to facilitate easy adsorption and desorption. Here, the authors report a composite catalyst where hydrogen adsorption occurs on rhodium and desorbs from silicon which has a lower adsorption energy.

## Graphical abstract

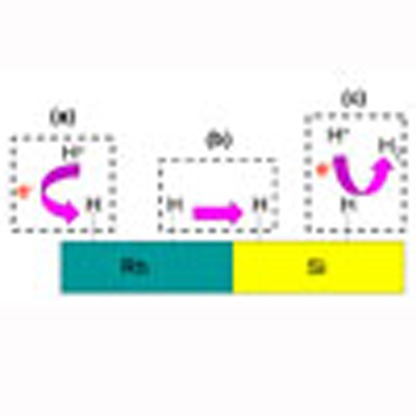

Hydrogen is considered to be one of the most important future sources of clean energy. Efficient and environmentally friendly methods for hydrogen production are thus extensively investigated. Electrolysis of water is one of the simplest and most advanced ways of hydrogen evolution^1^,^2^ which is currently being commercially used. This is why extensive research is still focused on the development of electrocatalysts with improved hydrogen evolution reaction (HER) efficiency.

A host of candidate materials for HER electrocatalytic applications were studied including metals^1^, sulfides^2^,^3^,^4^,^5^ and different composites (metals/sulfides^6^, sulfides/selenides^7^, C_3_N_4_/graphene^8^ and Co/graphene^9^). Platinum (Pt) is considered ‘the Holy Grail' of HER electrocatalysts, since Pt-based electrocatalysts have the best performance for hydrogen evolution, supplying fast kinetic rate and near-zero overpotential^7^,^10^.

One of the oldest rules in catalysis, Sabatier's principle^11^ states that for efficient HER the adsorption energy should be neither too high nor too low, because if it is too high (endothermic) adsorption is slow and the overall rate is slow as well. If it is too low (exothermic) desorption is slow^2^,^12^. To overcome the limitation of Sabatier's principle, we decided to separate the adsorbing surface and the desorbing surface by applying a metal/SiNW composite. Strong adsorption of hydrogen on the metal surface allows a large overall reaction rate, while high desorption from the SiNW surface allows a large evolution rate. We study several pure metals or metal/C composites as well as several metal/SiNW composites. The HER electrocatalytic activity of the metal/SiNW is found better than the pure metal electrode activity, supporting the validity of the composite concept for all metals investigated. More specifically, the HER activity of Rh/SiNW even surpasses that of Pt/C at sufficiently high overpotentials. Linear scan voltammetry (LSV) and hydrogen evolution measurements show that Rh/SiNW electrodes have a higher electricity-to-hydrogen-energy conversion efficiency than the commercial Pt/C electrodes. Moreover, at a high current density of 1,000 mA cm^−2^ the stability of hydrogen generation of the Rh/SiNW electrode is better than that of a Pt/C electrode. To support our experimental findings we also perform density functional theory (DFT) simulations of the Rh/SiNW system, which confirm the benefits of our composite approach of dividing the catalyst into two separate surfaces with large and small adsorption energies, respectively. The DFT calculations also show that Si poisoning by a hydroxyl can be removed by Rh-adsorbed hydrogen, which stabilizes the Rh/SiNW catalyst performance. This work thus opens the door for the design and fabrication of electrocatalysts with improved electric energy to hydrogen energy conversion efficiency.

## Results

### Structural characterization

LSV measurements were performed to probe the HER activity of the studied electrodes applying a typical three electrodes electrochemical workstation in an acidic medium (oxygen free 0.5 M H_2_SO_4_ serving as the electrolyte). The electrodes studied included commercial Pt/C electrodes (20 and 40 wt% Pt) representing the best currently available HER electrocatalyst. Several metal/SiNW electrodes were prepared with different concentrations of metals (denoted as *x* wt% M/SiNW with *x* being the relative mass of M in the composite).

**Figure 1:**
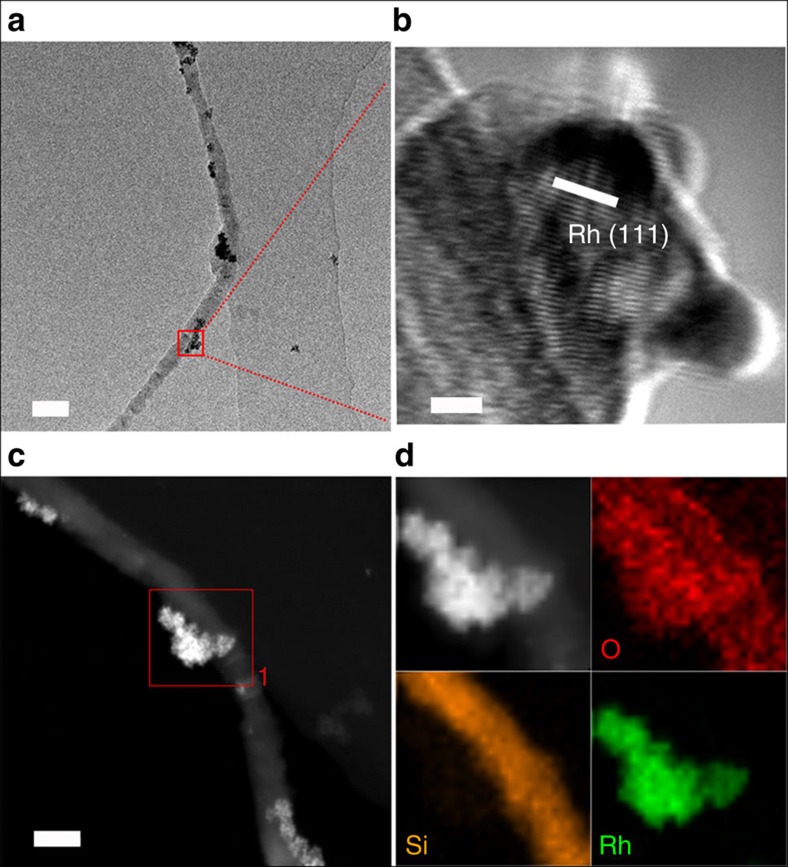
TEM characterization. (**a**) TEM image of a Rh/SiNW. Scale bar, 100 nm. (**b**) Enlarged HRTEM image of the red square in **a** showing a Rh crystallite. The 0.22 nm spacing corresponds to Rh (111). Scale bar, 5 nm. (**c**) HAADF-STEM image of a Rh/SiNW. Scale bar, 50 nm. (**d**) Its corresponding EDS mapping showing the O, Si and Rh distributions. EDS, energy dispersive spectroscopy; HAADF-STEM, high-angle annular dark field scanning TEM; HRTEM, high-resolution TEM.

SiNWs were prepared employing the oxide-assisted growth method^13^. Rh/SiNW was obtained by reducing Rh^3+^ ion via *in situ* growth of Rh nanoparticles (NPs) on the surface of hydrogen-terminated SiNWs^14^. All other M/SiNWs were obtained in a similar way. Supplementary Figs 1 and 2 are the X-ray diffraction pattern and X-ray photoelectron spectroscopy spectra of 29.1 wt% Rh/SiNWs, revealing their crystal structure and composition. Transmission electron microscopy (TEM) image (Fig. 1a) shows that the 29.1 wt% Rh/SiNW consists of a SiNW of 40 nm in diameter wrapped by Rh NPs. High-resolution TEM enlargement (Fig. 1b) of the red square in Fig. 1a shows a Rh NP embedded in the SiNW with the lattice spacing of 0.22 nm corresponding to the (111) interplanar distance of cubic Rh. High-angle annular dark field scanning TEM image of a single Rh NP attached to SiNW (Fig. 1c) and energy dispersive spectroscopy mapping show the elemental distribution of oxygen (red), silicon (orange) and rhodium (green), respectively (Fig. 1d).

### Electrochemical characterization

**Figure 2:**
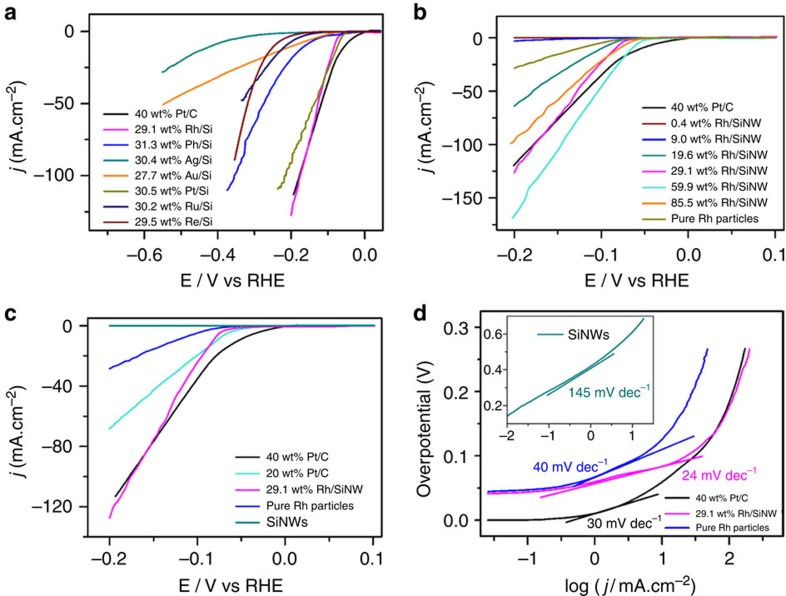
The electrochemical activity of different electrocatalysts in oxygen-free 0.5 M H_2_SO_4_. (**a**) LSV plots of metal/SiNW catalysts containing about 30 wt% metal. The metal type is indicated. The LSV of 40 wt% Pt/C is also shown. 29.1 wt% Rh/SiNW has the best HER activity of all metal/SiNW catalysts. (**b**) LSV plots of Rh/SiNW catalysts with different Rh wt% with the 59.9 wt% the best one. (**c**) LSV plots of SiNWs, pure Rh, 29.1 wt% Rh/SiNW, and 20 and 40 wt% Pt/C. The HER activity of 40 wt% Pt/C is better than that of 20 wt% Pt/C, so only the 40 wt% Pt/C data was used as a reference to the Rh/SiNW data. The HER activity of 29.1 wt% Rh/SiNW is worse than that of 40 wt% Pt/C for V>−160 mV but is better for V<−160 mV. (**d**) Tafel plots and Tafel slopes derived from **c**. The Tafel slopes are 145, 40, 30 and 24 mV dec^−1^ for SiNWs, pure Rh, 40 wt% Pt/C and 29.1 wt% Rh/SiNW, respectively. The Tafel slope of 29.1 wt% Rh/SiNW is smaller than that of 40 wt% Pt/C indicating the better HER performance of 29.1 wt% Rh/SiNW.

Figure 2a shows LSV curves of electrodes made of different metals/SiNW with a metal weight ratio of ∼30%. The best HER activities are obtained for Rh/SiNW and Pt/SiNW. Pt/SiNW has a slightly lower overpotential for a current density of 30 mA cm^−2^. The current increase with potential is however larger for Rh/SiNW than for Pt/SiNW so that the overpotential for 100 mA cm^−2^ is 0.18 V for Rh/SiNW compared with 0.22 V for Pt/SiNW. The LSV curves show the corresponding pure metals (Supplementary Fig. 3) to have a smaller HER activity than the M/SiNW composites validating that the composite concept of separating the adsorbing and desorbing site is indeed correct. Figure 2b presents the effect of the Rh concentration on the HER activity of Rh/SiNW catalysts, showing clearly that the HER activity for lower (0.4 and 9) wt% of Rh is worse than that of pure Rh NPs, whereas the HER activity for larger (19.6 and 29.1) wt% surpasses that of the pure Rh. The HER catalytic activity increases with increase in Rh content from 0.4 to 59.9 wt% and a further increment in Rh content (85.5 wt%) leads to decrease in the catalytic activity. This is due to the synergistic effect of adsorption on the Rh surface and desorption from the Si surface.

Figure 2c compares the LSV curves of pure SiNWs, pure Rh NPs, 20 and 40 wt% Pt/C (commercial catalysts) and 29.1 wt% Rh/SiNW. The 20 and 40 wt% Pt/C catalysts show nearly theoretical value (0 V versus RHE) for onset potential of hydrogen evolution. The 40 wt% Pt/C catalyst shows better HER characteristics than the 20 wt% Pt/C, so only the 40 wt% catalyst was subsequently used as a reference to the other catalysts. The 29.1 wt% Rh/SiNW and pure Rh NPs catalysts need 44 and 47 mV to sustain a current density of 0.1 mA cm^−2^. The SiNWs show negligible HER activity. It is interesting to note that the cathodic current of the 29.1 wt% Rh/SiNW-modified glassy carbon (GC) electrode increases with increasing overpotential much faster than that of the 40 wt% Pt/C. The exchange current densities of 29.1 wt% Rh/SiNW, pure Rh and 40 wt% Pt/C were calculated to be 0.00858, 0.0222 and 0.388 mA cm^−2^ (Supplementary Table 1). The 40 wt% Pt/C is the best one; the value ratio and Rh mass ratio of 29.1 wt% Rh/SiNW to pure Rh are 0.396 and 0.291, respectively, which indicates the exchange current density of 29.1 wt% Rh/SiNW is mainly from Rh active part.

**Figure 3:**
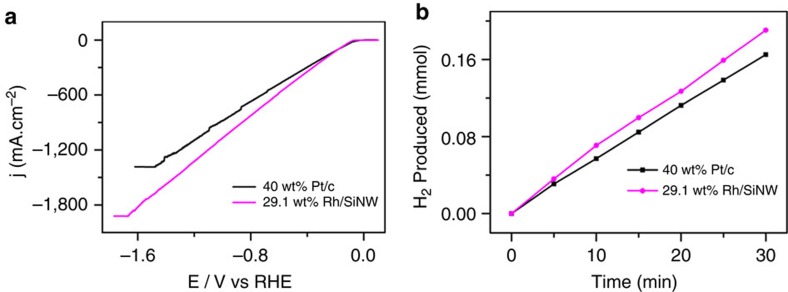
HER relative efficiency of 29.1 wt% Rh/SiNW and 40 wt% Pt/C in oxygen-free 0.5 M H_2_SO_4_. (**a**) LSV of both catalysts for high current densities. At 1,000 mA cm^−2^ the electric power to hydrogen energy conversion efficiency of 29.1 wt% Rh/SiNW is larger by 7.8%. Note that the current for 29.1 wt% Rh/SiNW is more stable. (**b**) Hydrogen evolution by 29.1 wt% Rh/SiNW and 40 wt% Pt/C systems at −0.4 V. The hydrogen evolution using 29.1 wt% Rh/SiNW is larger by 15%.

The cathodic current of 29.1 wt% Rh/SiNW catalysts thus exceeds that of the 40 wt% Pt/C ones when the potential is more negative than −160 mV (the current is 80 mA cm^−2^ at the intersection of these two curves). Neither SiNWs nor pure Rh catalysts show a better electrocatalytic performance than Pt/C. It is thus reasonable to deduce that the improved HER performance of the composite material is due to a synergistic electrocatalytic effect in the Rh/SiNW system. The Rh/SiNW catalysts are more advantageous than the conventional Pt/C electrodes for practical applications, since the production of hydrogen in industry requires current densities in the order of 1,000 mA cm^−2^ (ref. 15). The overpotentials of the 29.1 wt% Rh/SiNW nanocomposite and 40 wt% Pt/C catalyst are 0.95 and 1.12 V, respectively, at 1,000 mA cm^−2^ (Fig. 3a) with hydrogen to electric energy efficiencies of 56.4% and 52.3% (for details see Supplementary Note 1), respectively. Note that at high current densities, the LSV curve for the 29.1 wt% Rh/SiNW electrode is smooth and stable, whereas it is oscillatory and less stable for the 40 wt% Pt/C electrode.

To further substantiate the improved efficiency for hydrogen generation obtained by the Rh/SiNW system, we compared the hydrogen evolution of the 40 wt% Pt/C and the 29.1 wt% Rh/SiNW systems at the same potential of −0.4 V. Figure 3b shows that the hydrogen evolution of the Rh/SiNW system was larger by 15%.

### The synergistic HER reaction

The curves in Fig. 2c were re-plotted to get the Tafel plots and the Tafel slopes (Fig. 2d) to quantitatively compare the HER electrocatalytic activities.

The Tafel equation:


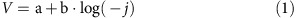


(where *V* is the overpotential, *j* the current density and b is the Tafel slope) is derived from the electrochemical kinetics, and reveals the relation between the electrochemical reaction rate and the overpotential. The Tafel slope is an important indicator in electrochemistry for the increase of the electrochemical current (that is, the reaction rate) upon an incremental increase of the overpotential. Small slopes represent a better electrocatalyst, since for small slopes the current gain with potential increase would be larger.

The Tafel slopes (Fig. 2d) for SiNW-, pure Rh- and Pt/C-modified GC electrodes derived from Fig. 2c are 145 (inset), 40 and 30 mV dec^−1^, respectively, whereas the Tafel slope of Rh/SiNW catalysts is as low as 24 mV dec^−1^. The Tafel slopes (Fig. 2d) show the 29.1 wt% Rh/SiNW to have the best HER kinetics among all electrocatalysts studied, since a smaller Tafel slope signifies a more rapid increase of the HER rate with increasing overpotential. The above discussion shows that Rh/SiNW not only has a catalytic activity comparable to Pt/C at low overpotentials (0 to −160 mV), but also is an excellent catalyst, even better than Pt/C in practical working potentials (more negative than −160 mV versus RHE).

There are three widely-accepted HER mechanisms.^16^

The Volmer mechanism:


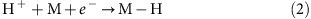


The Heyrovsky mechanism:


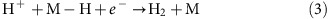


The Tafel mechanism:


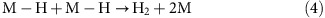


The theoretical Tafel slopes of the three mechanisms are 120, 40 and 30 mV dec^−1^, respectively^16^. Comparison of the experimental Tafel slopes with the theoretical values indicate that SiNWs, pure Rh and 40 wt% Pt/C catalysts follow the Volmer, Heyrovsky and Tafel mechanisms, respectively, while the Rh/SiNW catalyst (Tafel slope being 24 mV dec^−1^) may follow a different reaction mechanism.

**Figure 4:**
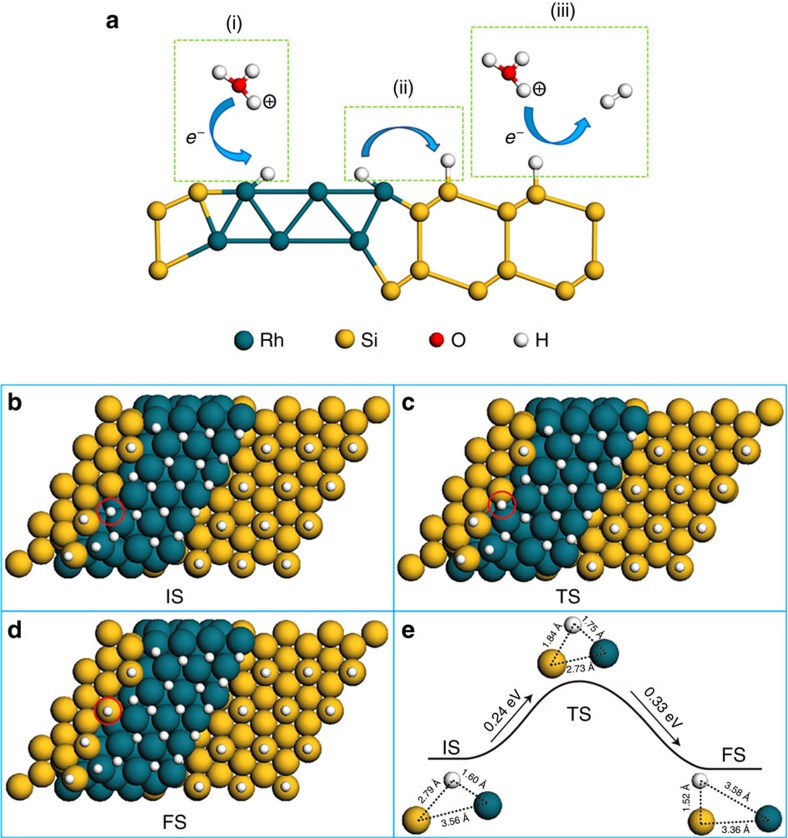
Modelling of the HER reaction on Rh/SiNW. (**a**) A schematic representation of the HER reaction mechanism: (i) adsorption of hydrogen ions on the Rh surface. (ii) Diffusion of a hydrogen atom from the Rh surface to the Si surface. (iii) The adsorbed hydrogen atom on the Si surface reacts with a proton to form a hydrogen molecule. And the Rh-adsorbed hydrogen may migrate to the surface of Si atoms (marked with a red circle): (**b**) IS, initial state; (**c**) TS, transition state; and (**d**) FS, final state. (**e**) The activation energy to get to the transition state is 0.24 eV and in the final state the adsorbed hydrogen is 0.33 eV below the transition state and 0.09 eV below the initial state.

We propose the following mechanism to explain the synergistic effect of the Rh/SiNW system. We suggest that the HER process in Rh/SiNW advances in three main steps (Fig. 4a): (i) adsorption of a hydrogen ion on the Rh surface (left part, Fig. 4a); (ii) migration of the neutral adsorbed-H atom to the Si surface (middle part, Fig. 4a); and (iii) reaction of the H atom with a hydrogen ion to produce a hydrogen molecule in the gas phase (right part, Fig. 4a).

The calculated adsorption free energy is also a key parameter to semi-quantitatively understand the variation in the exchange currents of catalysts^17^. DFT calculation was employed to obtain the difference of free energy (ΔG_H*_). The ΔG_H*_ of Rh/Si is −0.10 eV (Supplementary Table 4), which is very similar to the estimated on the Pt surface (−0.09 and −0.03 eV for 0.25 and 1 molecular layer, respectively)^17^.

DFT calculations were performed to substantiate the proposed mechanism. The Rh/SiNW catalyst was modelled with a hydrogen-terminated Si(111) surface and a Rh(111) surface (Supplementary Fig. 4a). The calculations indicate that in an acidic aqueous medium, the hydrogen ions quickly adsorb on the surface of Rh (Supplementary Fig. 4b) and obtain electrons to become atomic hydrogen atoms. The Rh-adsorbed hydrogen may then migrate to bare Si surface atoms near the Si/Rh interfaces (Figs 4b,c and Supplementary Movie 1). The calculated activation barrier of such a migration process is 0.24 eV per hydrogen atom, followed by an energy drop of 0.09 eV (Fig. 4e). The hydrogen adsorbed on the Si surface reacts with a hydronium ion (stabilized by water molecules in the solution) to produce a hydrogen gas molecule. This last reaction is a spontaneous process so that the rate-determining process is, therefore, the diffusion of atomic hydrogen from the Rh surface to the Si surface. Consequently the activation barrier of the entire HER process on Rh/SiNW is 0.24 eV, implying an excellent catalytic activity. Note that this activation barrier is much smaller than that on pure Rh catalysts (0.69 eV, Supplementary Fig. 5). Thus the synergistic HER process could be described by the following equations:


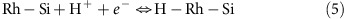



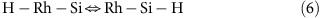



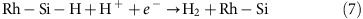


The detailed calculation of these reactions kinetics is given in the Supplementary Note 2.

The Tafel slope is: 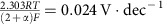 (assuming *α*=0.5, *F* is the Faraday constant, *R* the Rydberg gas constant and *T* the absolute temperature) in accord with the experimental data presented above.

Additional experiments provided further insight into the reaction mechanism. The pH-dependent relation of the HER was experimentally determined to be 2.13 (Supplementary Fig. 6), which is in accord with the theoretical value of 2 (Supplementary Note 2). The electrochemical impedance spectroscopy was also investigated (Supplementary Table 2 and Supplementary Fig. 7), a two-capacitive process supporting the formation of surface adsorption during the HER reaction. The electrochemical impedance spectroscopy was employed to calculate the real surface area and real current density (Supplementary Table 3) using Supplementary Methods.

The lifetime of a catalyst is an important parameter, especially when Si is used due to its poor stability in the oxidation environment. The Rh/SiNW and Pt/C catalysts were thus tested in oxygen-free 0.5 M H_2_SO_4_ at room temperature under a constant potential of −0.1 V showing that the cathodic currents remained steady for 500,000 s, indicating the long lifetime of both catalysts (Supplementary Fig. 8). The long lifetime of Rh/SiNW catalysts is supported by their self-regeneration ability, which was found by theoretical simulations and shown in Supplementary Fig. 9. When a Si surface atom is terminated with a hydroxyl (or in other words, poisoned by a hydroxyl), a Rh-adsorbed hydrogen may react with the hydroxyl to regenerate the Si atom by producing water molecules. The energy barrier of such water formation reaction is only 0.45 eV, and the energy of the final state is 0.06 eV lower than that of the initial one. The role of Rh in the regeneration of Si active sites is crucial to maintaining excellent electrocatalysis of the composite catalysts.

## Discussion

This work focuses on the improvement of the HER activity of electrocatalysts aiming to achieve better activity than that of Pt/C, which has the highest HER activity reported so far. We report an approach to overcome the limitations dictated by the Sabatier's principle (in which hydrogen adsorption and desorption occur on the same catalyst surface) by separating the adsorbing surface from the desorbing surface of HER catalysts. We design metal/SiNW composites as the catalysts in which the metal is the strongly hydrogen adsorbing surface and Si is the weakly adsorbing surface offering rapid evolution of hydrogen. We demonstrate that the approach indeed works, that is, the HER activity of the metal/SiNW composite is better than that of the pure metal. Rh/SiNW is shown to be an outstanding catalyst with HER activities exceeding those of Pt/C for sufficiently large current densities (high overpotentials) and with a Tafel slope of 0.024 V dec^−1^ compared with 0.03 V dec^−1^ for Pt/C. The lower Tafel slope of Rh/SiNW explains why the LSV curves of Rh/SiNW and Pt/C intersect, so that the HER activity of Rh/SiNW surpasses that of Pt/C for sufficiently high overpotentials (large current densities). Despite Pt/C showing lower overpotentials than Rh/SiNW catalysts at low current densities, the electric energy to hydrogen conversion efficiency of Rh/SiNW catalysts at high current densities of 1,000 mA cm^−2^ (as used by the industry) is 7.8% larger than that of Pt/C. The H_2_ generation rate of Rh/SiNW at the same overpotential of 0.4 V is 15% larger than that of Pt/C. Further, the Rh/SiNW electrode remained stable for continuous operation of 500,000 s (Supplementary Note 3).

The design concept of the Rh/SiNW electrode was checked and verified by theoretical calculations which provided insight into the HER mechanism and verified the low 0.024 V dec^−1^ Tafel slope obtained. The calculations support the regeneration of hydroxyl poisoning by Rh, which contributes to the long-term stability of the Rh/SiNW electrode. This work opens the door for a new way of producing improved electrocatalysts for HER overcoming theoretical limitations and potentially reducing the cost of hydrogen production by electrolysis.

## Methods

### Materials

RhCl_3_·3H_2_O was purchased from Aladdin Industrial Co.; Nafion (5 wt%), 20 and 40% Pt/C catalysts from Sigma-Aldrich Co. Other reagents used were of analytical reagent grade without further purification. Doubly distilled water was used throughout the experiment.

### Fabrication of metal/SiNW catalysts

Silicon nanowires obtained via oxide-assisted growth method^13^ were etched with 5 ml 5% HF aqueous solution for 1 min to remove the SiO_2_ sheath and to form Si–H bonds on their surface. The etched-SiNWs were rinsed with distilled water, and then immersed in 10 ml of different concentrations of RhCl_3_ aqueous solution with stirring for 30 min to form Rh/SiNW with different contents of Rh. 29.1 wt% Rh/SiNW was immersed in excessive 5% HF aqueous solution to etch the residual silicon nanowires to ultimately obtain pure Rh NPs. These NPs were used to study the electrochemical properties of pure Rh. Ag/SiNW nanocomposites was prepared by reducing silver nitrate, while (Pd, Au, Pt, Ru and Re)/Si nanocomposites were obtained by reducing corresponding metal chloride aqueous solutions in a similar manner at the appropriate concentrations. 30.5 wt% Pt/SiNW was immersed in excessive 5% HF aqueous solution to etch the residual SiNWs to ultimately obtain pure Pt NPs.

### Characterization

The structure of the samples was characterized by X-ray diffraction (Philips X'pert PRO MPD diffractometer) applying Cu Kα radiation (*λ*=0.15406, nm). TEM and high-resolution TEM images were recorded using a FEI Tecnai F20 transmission electron microscope with accelerating voltage of 200 kV. The chemical states of the catalysts were studied by X-ray photoelectron spectroscopy performed using a Kratos AXIS UltraDLD ultrahigh vacuum surface analysis system with Al Kα radiation (1486, eV) as probe. The Brunauer–Emmett–Teller (BET) specific surface area of these samples was investigated by the ASAP 2020 instrument at 77 K. The Rh and Si contents of the catalysts were determined with Hitachi 180-80 atomic absorption spectrometer and ND-2105 trace silicate analyser. The hydrogen gas content was detected with a gas chromatograph (GC-7890T) with nitrogen as carrier gas.

### Electrochemical measurements

The electrochemical measurements were carried out in a conventional three-electrode cell connected to a Princeton VersaSTAT4 electrochemistry workstation. A platinum plate with an area of 2 cm^2^ was used as the counter electrode, a saturated calomel electrode (SCE) was used as the reference electrode and a modified GC electrode with a diameter of 0.3 cm, into which the studied catalysts were introduced, as the working electrode. The area of the GC electrode was calculated to be 0.0707, cm^2^, which was used to determine the current density in LSV. The working electrode was installed facing upward for the quick release of the evolving hydrogen gas.

For a typical fabrication of the working electrode, 2 mg catalysts were dispersed in 1 ml of 5:1 v/v water-isopropanol mixed solvent with 100 μl 0.5 wt% Nafion solution, and ultrasonically stirred for at least 30 min to reach a homogeneous suspension. Then 7.5 μl of the suspension was loaded onto a GC electrode with a diameter of 3 mm (loading ∼0.193 mg cm^−2^). Finally, the as-prepared catalyst film was dried at room temperature.

The potentials reported in this work were versus RHE applying the calibration procedure described below. In all measurements a SCE electrode was used as the reference and the potential values were calibrated with respect to RHE. The calibration was performed in a high-purity hydrogen-saturated electrolyte with a Pt foil as the working electrode. In 0.5 M H_2_SO_4_ solution, E_vs.RHE_=E_vs.SCE_+0.251 V. All the potentials reported in the manuscript were against RHE. Before any electrochemical measurement, the electrolyte solution was purified with N_2_ to completely remove oxygen, and stable polarization performance was recorded after 10 cycles. All data were reported without iR compensation.

### Calculations

DFT calculations were carried out with the VASP code^18^,^19^. The electron–ion interactions were described with the projector augmented wave method^20^ and an energy cutoff of 400 eV. The GGA-PBE functional^21^ were employed to describe the exchange-correlation energy. The effect of van der Waals interactions was included with the van der Waals-DF method^22^. The Si(111) and Rh(111) surfaces were represented using periodic slabs, consisting of six Si and four Rh atomic layers, respectively. The bottom four layers of Si and two layers of Rh atoms were kept fixed. Structural relaxations were stopped when the force on each atom was smaller than 0.01 eV per Angstrom. The transition states were determined with the climbing image nudged-elastic band method. Five structural images were inserted between the initial states and the final states in all climbing image nudged-elastic band calculations. The first Irreducible Brillouin zone was sampled using a 2 × 2 × 1 Monkhorst-Pack grid for all calculations.

In the proposed mechanism, the hydrogen atoms on Si atoms are coming from the Rh surface rather than from the solution. Correspondingly, the difference of free energy (ΔG_H*_) is calculated by ΔG_H*_=ΔE_H_+ΔE_ZPE_-TΔS=G_H-Si/Rh_– G_H-Rh/Si_. Here, G_H-Rh/Si_ represents the free energy when the hydrogen atom is adsorbed on a Rh surface at the Rh/Si interface (see Fig. 4b), while G_H-Si/Rh_ represents the free energy when the adsorbed hydrogen is diffused on a Si atom at the Rh/Si interface (see Fig. 4d). Taking into consideration that the vibrational entropy in the adsorbed state is very small^17^, the contribution from entropy change (TΔS) is neglected. The ΔE_H_ and ΔE_ZPE_ are obtained from our DFT calculations. The calculated ΔG_H*_=ΔE_H_+ΔE_ZPE_=−0.10 eV. This value is also listed in Supplementary Table 2.

### Data availability

All relevant data are available on request, which should be addressed to M.S.

## Additional information

**How to cite this article:** Zhu, L. *et al*. A rhodium/silicon co-electrocatalyst design concept to surpass platinum hydrogen evolution activity at high overpotentials. *Nat. Commun.* 7:12272 doi: 10.1038/ncomms12272 (2016).

## Supplementary Material

Supplementary Information Supplementary Figures 1-9, Supplementary Tables 1-4, Supplementary Notes 1-3, Supplementary Methods and Supplementary References.

Supplementary Movie 1 The Rh-adsorbed hydrogen may migrate to bare Si surface atoms near the Si/Rh interfaces, which help to enhance the electrocatalytic ability for hydrogen evolution reaction.
