# Supplementary material for: A rhodium/silicon co-electrocatalyst design concept to surpass platinum hydrogen evolution activity at high overpotentials
Source: Nat Commun. 2016 Jul 22;7:12272. doi: 10.1038/ncomms12272 (PMC4961846; doi:10.1038/ncomms12272)
Supplement: Supplementary Information — Supplementary Figures 1-9, Supplementary Tables 1-4, Supplementary Notes 1-3, Supplementary Methods and Supplementary References. [file ncomms12272-s1.pdf]

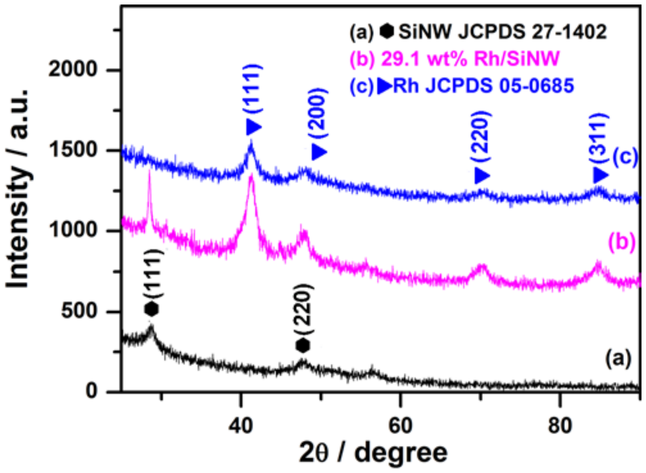

Supplementary Figure 1. XRD patterns of (a) SiNWs, (b) Rh/SiNW, and (c) Rh nanoparticles.

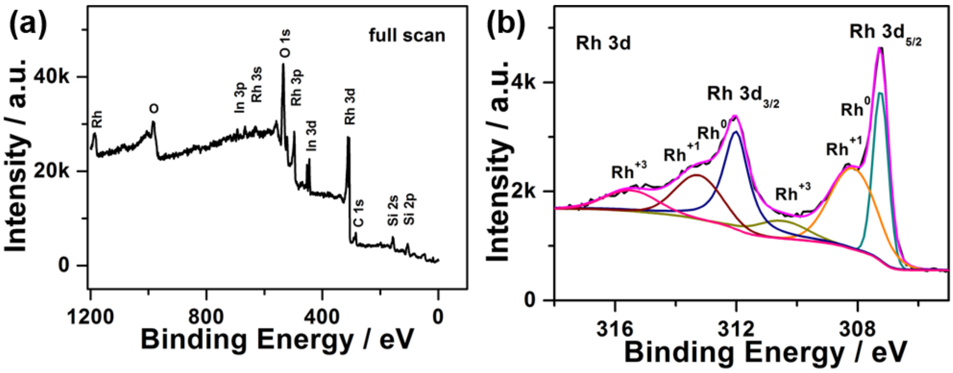

Supplementary Figure 2. XPS spectra of Rh/SiNW: (a) full spectrum, and (b) Rh 3d spectrum, using an indium plate as the supporter of the powder samples.

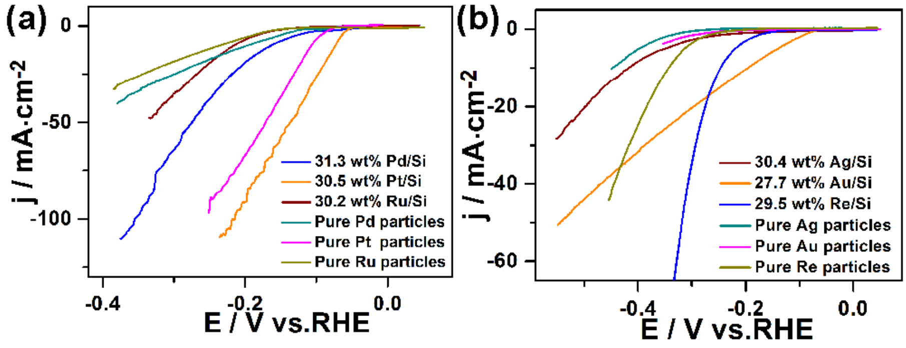

Supplementary Figure 3. LSV curves of various catalysts: (a) 31.3 wt% Pd/Si, 30.5 wt% Pt/Si, 30.2 wt% Ru/Si, pure Pd, pure Pt, pure Ru; and (b) 30.4 wt% Ag/Si, 27.7 wt% Au/Si, 29.5 wt% Re/Si, pure Ag, pure Au, pure Re, showing that the catalytic activities of metal/SiNW catalysts are better than those of metal ones.

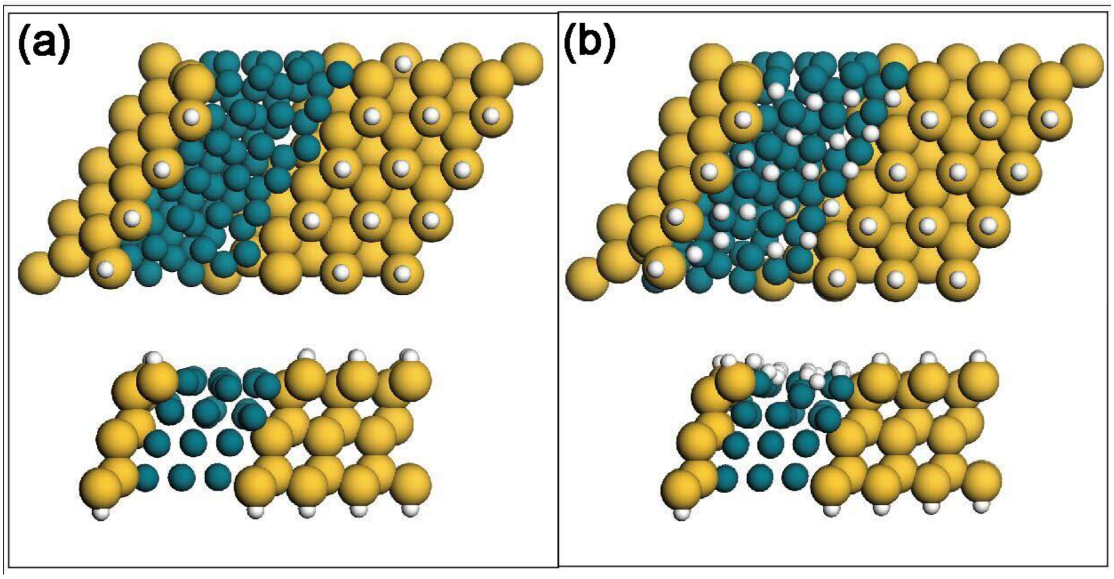

Supplementary Figure 4. Optimized structure of Rh/Si composite and the adsorption of hydrogen atoms on the Rh: (a) The optimized structure of Rh/Si composite catalysts; and (b) the adsorption of H atoms on the Rh. Upper: top view; lower: side view. The adsorption of hydrogen atoms on the Rh is easy and with a negligible energy barrier. The H^+ ion adsorbs above an on-top site in the first step, and then diffuses to an hcp hollow site.

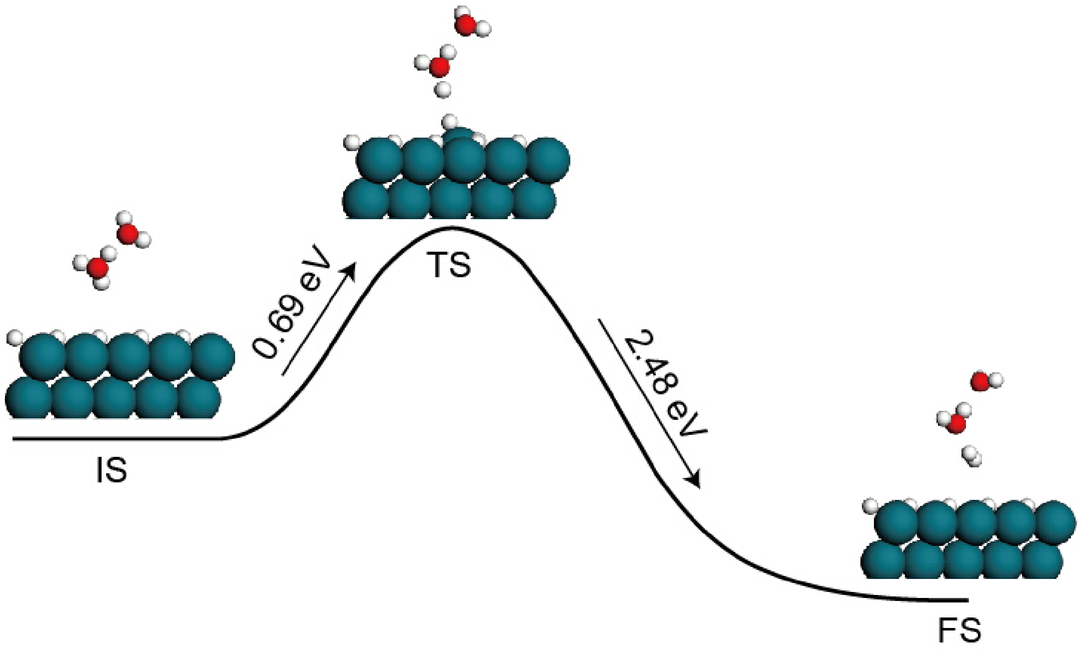

Supplementary Figure 5. Reaction pathway of HER on pure Rh catalysts: IS, initial state; TS, transition state; and FS, final state. The proton in acidic media is stabilized with two water molecules. The entire system has a positive charge of +1. The activation barrier of this process is calculated to be 0.69 eV with an energy drop of 1.79 eV via DFT simulation.

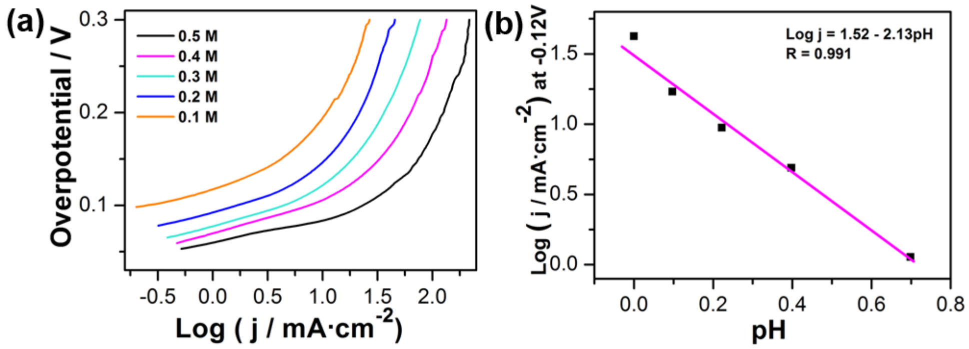

Supplementary Figure 6. pH-dependent HER reaction order: (a) A typical plot of overpotential versus log j using 29.1 wt% Rh/SiNW in oxygen-free xM H_2SO_4 (x = 0.1, 0.2, 0.3, 0.4, and 0.5 M) at a sweep rate of 5 mV·s^{-1}; and (b) the pH dependence of the cathodic current density of HER at −0.12 V vs. RHE, showing the reaction order of 2.13, which is close to the theoretical value of 2 in Equation 5.

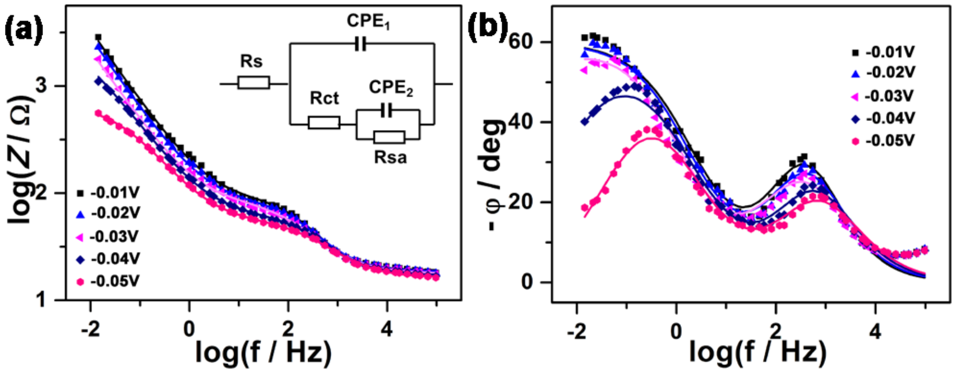

Supplementary Figure 7. Electrochemical impedance spectroscopy using 29.1 wt% Rh/SiNW in the frequency ranging between 100 kHz to 100 mHz with overpotentials of 0.01 to 0.05 V: (a) Bode magnitude plot and (b) Bode phase plot of Rh/SiNW. Symbols, experimental data; solid lines, fitted data. The inset shows the equivalent circuit model. A modelwas applied to be fitted to the impedance spectra of Rh/SiNW electrodes.

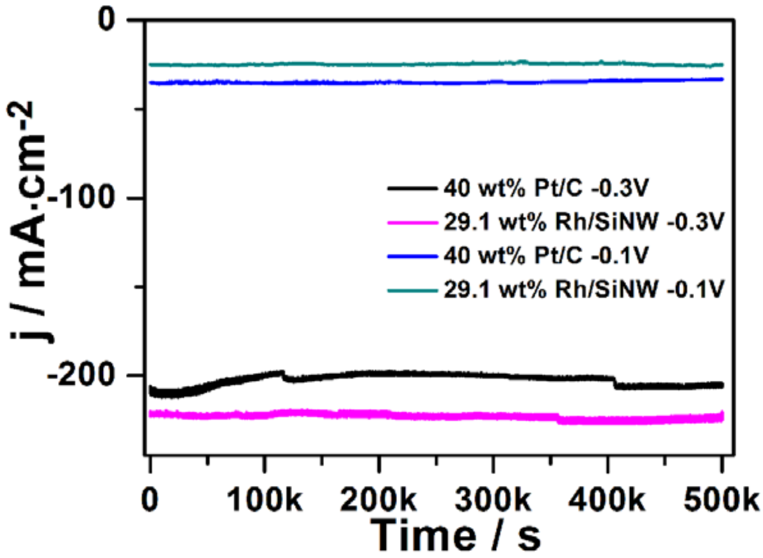

Supplementary Figure 8. Stability experiment: I-t curves of 29.1 wt% Rh/SiNW and 40 wt % Pt/C catalysts under -0.1 or -0.3 V in oxygen-free 0.5 M H_2SO_4.

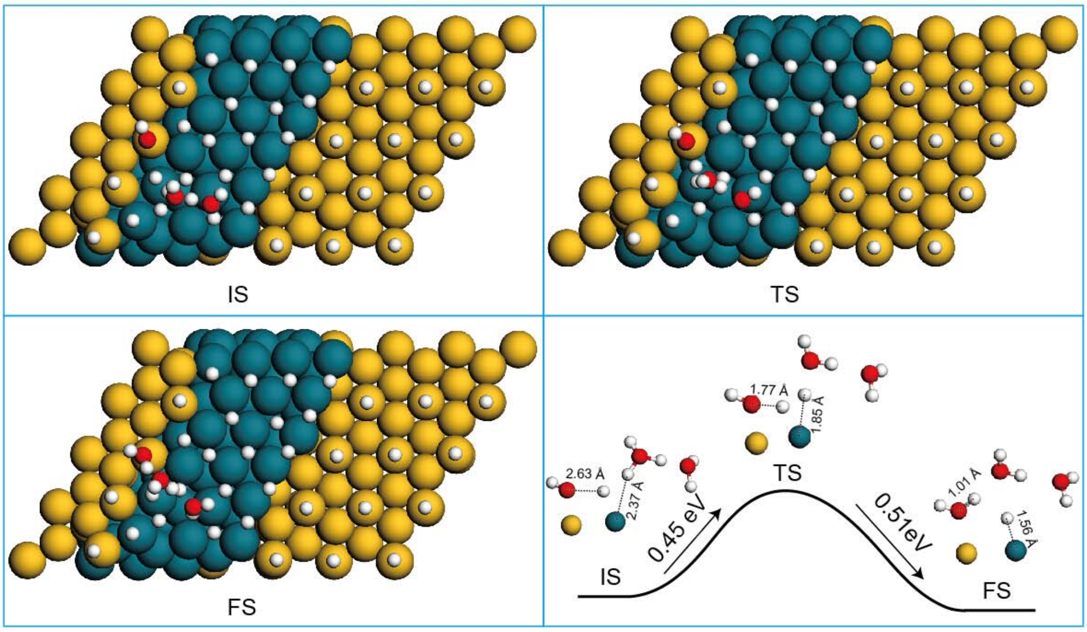

Supplementary Figure 9. Stability calculation: The reaction pathway of removal of the hydroxyl from the Si surface by the formation of a water molecule. The H atom may migrate from the Rh(111) surface to the neighboring Si surface and react with the adsorbed hydroxyl to produce a water molecule. The calculated activation barrier is 0.45 eV: IS, initial state; TS, transition state; and FS, final state.

Suppmenentary Table 1. Their exchange current density of various electrocatalysts

	$j_{o,app}$, mA·cm^{-2}
40wt% Pt/C	0.388
29.1wt% Rh/SiNW	0.00858
31.3wt% Pd/SiNW	0.308
30.4wt% Ag/SiNW	0.00862
27.7wt% Au/SiNW	0.00617
30.5wt% Pt/SiNW	0.0377
30.2wt% Ru/SiNW	0.00960
29.5wt% Re/SiNW	0.00528
Pure Rh	0.0222
SiNW	0.00140

Supplementary Table 2. Numerical values of the parameters of the equivalent circuits for the 29.1 wt% Rh/SiNW electrocatalyst during HER

Potential, V	-0.01	-0.02	-0.03	-0.04	-0.05
R_s, Ω	18.96	17.67	17.00	16.22	15.85
CPE_1, Y_{01}					
$S \cdot sec^{n1}$	0.0000736	0.000183	0.001178	0.0001532	0.0001838
n1	0.7751	0.6726	0.7116	0.6787	0.6500
R_{ct}, Ω	71.49	68.93	46.72	40.23	33.89
CPE_2, Y_{02}					
$S \cdot sec^{n2}$	0.001868	0.002785	0.003063	0.003519	0.004773
n2	0.6651	0.6699	0.6451	0.6628	0.6496
R_{sa}, Ω	5.11E+14	3.65E+04	3.54E+03	6.32E+02	2.98E+02

Supplementary Table 3. Cdl values of the different catalysts and their exchange current density

	C_{dl}, F	S_{real},·cm^2	$j_{0,real}$, mA·cm^{-2}
40wt% Pt/C	4.289E-5	2.14	0.0128
29.1wt% Rh/SiNW	6.604E-6	0.330	0.00184
31.3wt% Pd/SiNW	3.424E-5	1.71	0.0127
30.4wt% Ag/SiNW	6.663E-6	0.333	0.00183
27.7wt% Au/SiNW	2.435E-6	0.122	0.00357
30.5wt% Pt/SiNW	2.768E-5	1.38	0.00193
30.2wt% Ru/SiNW	2.481E-5	1.24	5.47E-4
29.5wt% Re/SiNW	1.959E-6	0.0980	0.00381
Pure Rh	1.745E-5	0.872	0.00180
SiNW	2.317E-7	0.0116	0.00851

*Sapp - apparent work electrode area; Sreal - real work electrode area; $j_{0,app}$ - apparent exchange current density; $j_{0,real}$ - real exchange current density, C_{dl} and S_{real} were calculated according to the Equations (6) and (7).

Supplementary Table 4. Calculated hydrogen adsorption energy ΔE_H and measured exchange currents j_0

	ΔG_{H*}(eV)	ΔG_{H*}(eV)	*Log(j_o,* mA·cm^2)
Pt	-0.09 (0.25 ML)[1]	-0.03 (1 ML)[1]	-1.893
Rh/Si	-0.10		-2.735

Supplementary Note 1

The current in industrial electrochemical hydrogen production may reach the order of 1000 mA·cm $^{-2}$. The LSV curves for 29.1 wt% Rh/SiNW and 40 wt% Pt/C catalysts were recorded in oxygen-free 0.5 M H_2SO_4 at room temperature, showing the overpotentials of 0.95 and 1.12 V, respectively.

These values may be used to estimate the electric to hydrogen energy conversion efficiency of the catalysts in industrial electrochemical hydrogen production according to the following equation:

$\eta = \frac{1.23 \times I}{(1.23 + V) \times I} \times 100\% = \frac{1.23}{1.23 + V} \times 100\%$, where *V* is the overpotential.

Therefore, the efficiencies of the 29.1 wt% Rh/SiNW and the 40 wt% Pt/C catalysts are $\eta_{Rh/Si} = \frac{1.23}{1.23 + 0.95} \times 100\% = 56.4\%$ and $\eta_{Pt/C} = \frac{1.23}{1.23 + 1.12} \times 100\% = 52.3\%$, respectively. The efficiency of the Rh/SiNW catalyst is larger by 7.8% ($\frac{\eta_{Rh/Si} - \eta_{Pt/C}}{\eta_{Pt/C}} \times 100\%$) than that of Pt/C.

The BET surface areas of 29.1 wt% Rh/SiNW and 40 wt% Pt/C are 71.8 and 137.7 $m^2 \cdot g^{-1}$ using N_2 adsorption–desorption isotherms.

Supplementary Note 2

Based on the simulation, a possible path is proposed to explain the synergistic electrocatalysis of Rh and Si.

$$Rh-Si+H^{+}+e^{-}\Leftrightarrow H-Rh-Si \quad (1)$$

$$H-Rh-Si\Leftrightarrow Rh-Si-H \quad (2)$$

$$Rh-Si-H+H^{+}+e^{-}\rightarrow H_{2}+Rh-Si \quad (3)$$

The kinetics may be complicated. The reaction velocity of hydrogen evolution may be written as $r=k_{3}\theta_{Si-H}C_{H^{+}}$, where r is the reaction rate; k the rate constant; θ the fractional occupancy of the H-adsorption sites; and $C_{H^{+}}$ the concentration of hydrogen ion.

In the steady state, for θ_{Si-H},

$$\frac{d\theta_{Si-H}}{dt}=k_{2}\theta_{Rh-H}(1-\theta_{Si-H})-k_{-2}\theta_{Si-H}(1-\theta_{Rh-H})-k_{3}\theta_{Si-H}C_{H^{+}};$$

and for θ_{Rh-H}

$$\frac{d\theta_{Rh-H}}{dt}=k_{1}(1-\theta_{Rh-H})C_{H^{+}}-k_{-1}\theta_{Rh-H}-k_{2}\theta_{Rh-H}(1-\theta_{Si-H})+k_{-2}\theta_{Si-H}(1-\theta_{Rh-H}).$$

At low overpotential,

$$\theta_{Si-H}\approx\frac{k_{2}\theta_{Rh-H}}{k_{2}\theta_{Rh-H}+k_{-2}-k_{-2}\theta_{Rh-H}+k_{3}C_{H^{+}}}\approx\frac{k_{20}}{k_{-20}}\theta_{Rh-H}e^{-\frac{F\Delta\varphi}{RT}}$$

$$\theta_{Rh-H}\approx\frac{k_{1}C_{H^{+}}+k_{-2}\theta_{Si-H}}{k_{1}C_{H^{+}}+k_{-1}+k_{2}+k_{-2}\theta_{Si-H}-k_{2}\theta_{Si-H}}\approx\frac{k_{10}}{k_{-10}}C_{H^{+}}e^{-\frac{F\Delta\varphi}{RT}}$$

Then, $r=k_{3}\theta_{Si-H}C_{H^{+}}=\frac{k_{10}k_{20}k_{30}}{k_{-10}k_{-20}}C_{H^{+}}^{2}e^{-\frac{(2+\alpha)F\Delta\varphi}{RT}}$ (4)

And $-j=Fr=\frac{k_{10}k_{20}k_{30}}{k_{-10}k_{-20}}FC_{H^{+}}^{2}e^{-\frac{(2+\alpha)F\Delta\varphi}{RT}}$

$$\lg(-j) = Const + 2\lg C_{H^+} - \frac{(2+\alpha)F}{2.303RT}\Delta\varphi \qquad (5)$$

Therefore, the Tafel slope is: $\frac{2.303RT}{(2+\alpha)F}$ = 0.024 V·dec^{-1} (assuming $\alpha = 0.5$)

Supplementary Note 3

The electrolyte were also analyzed using atomic absorption spectroscopy and trace silicate analyzer, after HER was conducted at -0.1 or -0.3 V at room temperature for 500,000 s. The results show that the concentrations of Rh and Si in the electrolyte are below the detection limit of the equipment (less than 1 $ng\cdot L^{-1}$ and 1 $\mu g\cdot L^{-1}$, respectively), indicating no corrosion of the Rh/SiNW catalysts.

Supplementary Methods

The equivalent circuit in electrochemical impedance spectroscopy is represented by the solution resistance R_s connected in series with three other parts: the resistance of the charge-transfer step R_{ct}, the double layer capacitance C_{dl}, and resistance R_{sa} connected in series with capacitance C_{sa}; and these three parts are connected in parallel. The double layer capacitance C_{dl} and capacitance C_{sa} were replaced by constant phase elements CPE_1 and CPE_2. The resistance R_{sa} and capacitance C_{sa} were employed to describe the formation of surface adsorption during the HER reaction. There emerging peak in the Bode plots indicates that an additional capacitive element is produced.

C_{dl} and S_{real} were calculated according to the following equations:[2, 3]

$$C_{dl} = (Y_{01} / (R_s^{-1} + (R_{ct} + R_{sa})^{-1})^{1-n1})^{\frac{1}{n1}}; \qquad (6)$$

$$S_{real} = \frac{C_{dl}}{k}, k = 20\mu F / cm^2 \qquad (7)$$

Supplementary References

1. Nørskov, J. K. *et al.* Trends in the exchange current for hydrogen evolution, *J. Electro. Soc.* **152**, J23-J26 (2005).

2. Herraiz-Cardona, I.; Ortega, E.; Pérez-Herranz, V. Impedance study of hydrogen evolutionon Ni/Zn and Ni-Co/Zn stainless steel based electrodeposits. *Electrochim. Acta* **56,** 1308-1315 (2011).

3. Lasia, A. *Electrochemical Impedance Spectroscopy and its Applications* P. 220 (Springer Science-Business Media, New York, 2014).
