# Supplementary figures and images for: A rhodium/silicon co-electrocatalyst design concept to surpass platinum hydrogen evolution activity at high overpotentials
Source: Nat Commun. 2016 Jul 22;7:12272. doi: 10.1038/ncomms12272 (PMC4961846; doi:10.1038/ncomms12272)

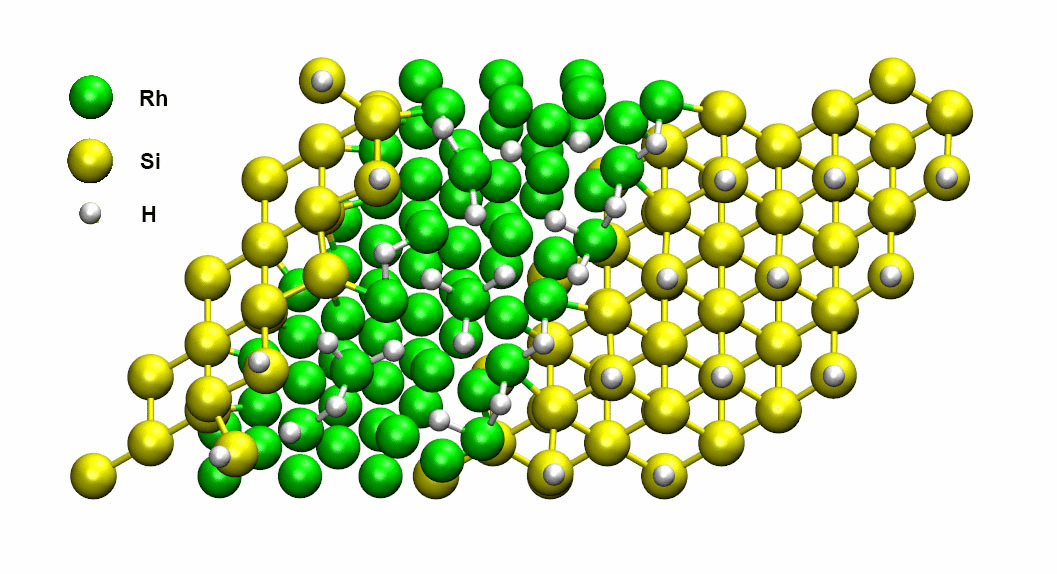

Supplement: Supplementary Movie 1 — The Rh-adsorbed hydrogen may migrate to bare Si surface atoms near the Si/Rh interfaces, which help to enhance the electrocatalytic ability for hydrogen evolution reaction. [file ncomms12272-s2.gif]
